# Knockout of *MARCH2* inhibits the growth of HCT116 colon cancer cells by inducing endoplasmic reticulum stress

**DOI:** 10.1038/cddis.2017.347

**Published:** 2017-07-27

**Authors:** Dan Xia, Wanli Ji, Chentong Xu, Xin Lin, Xiaokun Wang, Yan Xia, Ping Lv, Quansheng Song, Dalong Ma, Yingyu Chen

**Affiliations:** 1 Department of Immunology, Peking University School of Basic Medical Science, Key Laboratory of Medical Immunology, Ministry of Health, Peking University Health Sciences Center, 38 Xueyuan Road, Beijing 100191, China; 2 Department of Pathology, Shandong Medical College, 6 Jucai Road, Linyi 276000, China; 3 Center for Human Disease Genomics, Peking University, 38 Xueyuan Road, Beijing 100191, China; 4 Department of Pathology, Affiliated Hospital of Shandong Medical College, 80 Jintan Road, Linyi 276000, China

## Abstract

Membrane-associated RING-CH protein 2 (MARCH2), a member of the MARCH family, functions in vesicle trafficking and autophagy regulation. In this study, we established *MARCH2* knockout HCT116 cell lines using CRISPR/Cas9-mediated genome editing to evaluate the role of MARCH2 in colon cancer *in vitro* and *in vivo*. Knockout of *MARCH2* suppressed cell proliferation, and promoted autophagy, apoptosis and G2/M phase cell cycle arrest. These effects were associated with activation of endoplasmic reticulum (ER) stress. In addition, loss of *MARCH2* sensitized HCT116 cells to the chemotherapy drugs etoposide and cisplatin. Moreover, we analyzed the clinical significance of MARCH2 in human colon carcinoma (*n*=100). High MARCH2 expression was significantly associated with advanced clinicopathological features and poorer overall survival in colon carcinoma. MARCH2 expression correlated negatively with expression of the unfolded protein response molecule p-PERK in colon cancer. Collectively, these data reveal a relationship between MARCH2, ER stress and colon cancer, and indicates MARCH2 may have an important role in the development and progression of colon cancer.

The endoplasmic reticulum (ER) is a cellular membrane compartment in eukaryotic cells involved in the synthesis and sorting of secretory and membrane proteins that is also an important site for initiation of autophagosome formation.^1, 2, 3^ Many different perturbations can alter the function of ER, leading to de accumulation of unfolded or misfolded proteins inside the ER, a cellular referred to as ER stress. ER stress initiates a series of adaptive mechanism that together are known as the unfolded protein response (UPR).^3^

The ER stress is a complex process that involves activation of three major signaling pathways: inositol-requiring enzyme-1*α* (IRE1*α*), activating transcription factor 6 (ATF6) and protein kinase RNA-like endoplasmic reticulum kinase (PERK). In response to the accumulation of unfolded proteins in the ER, GRP78/BiP is released from IRE1*α*, ATF6 and PERK to chaperone the accumulated proteins to degradation via ubiquitination.^4^ GRP78/BiP-free ER stress sensors are differentially activated to initiate their downstream cascades. Activated IRE1*α* acts as an RNase to process the mRNA encoding XBP1, leading to the expression of an active transcription factor (XBP1s, s correspond to splicing). XBP1s functions as a transcriptional activator for UPR gene targets such as GRP78/BiP and calreticulin.^5, 6^ Concomitantly, during ER stress, ATF6*α* is released from GRP78/Bip and translocates from the ER to Golgi where it undergoes cleavage. Cleaved ATF6*α* translocates to the nucleus and transactivates various chaperones and major ER stress markers such as the CAAT-enhancer binding protein (CHOP) gene.^6^ Moreover, increased expression of CHOP has been reported to activate apoptosis in various studies.^7^

The PERK/EIF2*α* pathway is a component of the UPR signaling pathway: when no ER stress is present, PERK is combined with GRP78/Bip in an inactive state; under ER stress conditions, PERK separates from its molecular chaperone GRP78/Bip and becomes activated, and phosphorylates and inactivates EIF2*α* leading to termination of the majority of cellular protein synthesis, which in turn regulates the cell cycle. The PERK/ EIF2*α* pathway also activates ATF4, which upregulates CHOP expression.^8^ CHOP is a specific transcription factor of ER stress, which induces the expression of the ER stress-related protein CKI and genes related to cell cycle regulation.^9^

Membrane-associated RING-CH protein 2 (MARCH2), contains a RING domain that exerts E3 ubiquitin ligase activity.^10^ MARCH2 was first described as a member of the ubiquitin ligase family probably related to viral immune evasion proteins.^11^ MARCH2 participates in vesicle trafficking by interacting with syntaxin 6.^12^ As an E3 ubiquitin ligase, MARCH2 can ubiquitinate several substrates, such as DLG1,^13^ *β*2AR^14^ and CFTR.^15^ In our previous study, we demonstrated that MARCH2 negatively regulates cell autophagy.^16^ This mechanism involves inhibition of the PIK3CA-AKT-MTOR and CFTR signaling pathways. However, the role of MARCH2 in tumor remains unclear.

Clustered regularly interspaced short palindromic repeats/Cas9 (CRISPR/Cas9) is a newly developed biotechnology for editing genomic DNA. The CRISPR/Cas9 system consists of two components: a single guide RNA (sgRNA) that provides target specificity and a CRISPR-associated protein (Cas9) that functions as a helicase and endonuclease.^17^ Cas9 unwinds and cleaves double-stranded DNA containing the protospacer adjacent motif (NGG; N indicates any base) through the sgRNA.^18^ The non-homologous end-joining DNA repair pathway is then activated to repair the lesion, generating errors that eventually interrupt the open reading frame and lead to dysfunctional gene expression at the transcriptional level.^19^

In this paper, we demonstrate that knockout of *MARCH2* using CRISPR/Cas9 gene editing biotechnology suppressed the growth of colon cancer cells *in vitro* and *in vivo* via effects associated with the ER stress pathway.

## Results

### Knockout of *MARCH2* using CRISPR/Cas9-mediated genome editing inhibits cell proliferation

**Figure 1:**
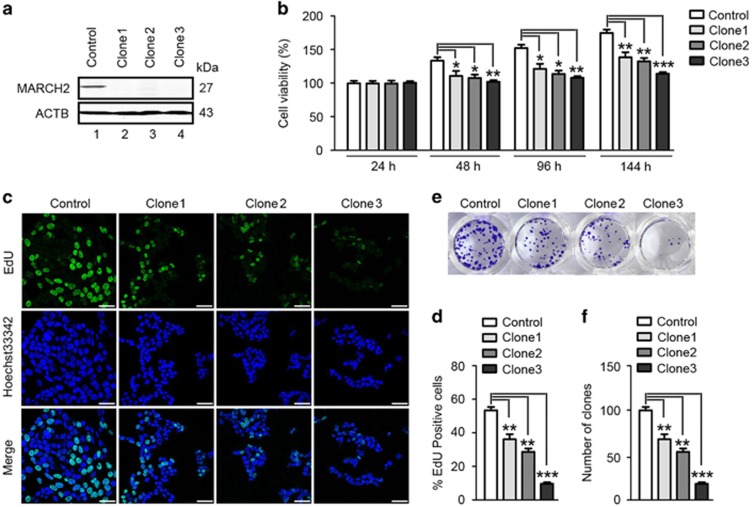
Knockout of *MARCH2* suppresses colon cancer cell growth. (**a**) Western blot analysis of MARCH2 protein expression in Cas9-*MARCH2* HCT116 cells. (**b**) MTS cell viability assay. Control (wild-type) and Cas9-*MARCH2* HCT116 cells were seeded in 96-well plates (3000 cells/well; five replicates), serum-starved for 18 h and then pulsed with 10% FCS for 24 h, 48 h, 96 h or 144 h. Data are mean±S.D. of three independent experiments. (**c**) Representative confocal microscopy of immunofluorescent staining for EdU. Control and Cas9-*MARCH2* HCT116 cells were plated on glass slides in 24-well plates, serum-starved for 18 h, pulsed with 10% FCS for 48 h and incubated with EdU for 4 h. Nuclei were stained with Hoechst 33342. Scale bar: 100 mm. (**d**) Quantification of the percentage of EdU-positive cells (in 200 cells). Each bar represents the mean±S.D. of three independent experiments. (**e**) Representative images of colony formation by control (wild-type) cells and Cas9-*MARCH2* HCT116 cells. (**f**) Quantitative analysis of colony numbers for three independent experiments. *P<0.05, **P<0.01, ***P<0.001

To clarify the function of MARCH2 in colon cancer, we knocked out *MARCH2* in HCT116 colon cancer cells. Through a series of screens, three Cas9-*MARCH2* clones were selected. Sequence analysis revealed the three clones, clone 1, GT→GCT; clone 2, AGGTCG→AG; clone 3, TCGTGG→C, contained in-frame shift mutations which disrupted the *MARCH2* ORF, leading to deletion of the transmembrane, RING or PDZ functional domains (Supplementary Figure 1a–c). Western blotting indicated MARCH2 protein was not detectable in Cas9-*MARCH2* HCT116 cells (Figure 1a).

Next, we tested the viability of the Cas9-*MARCH2* HCT116 cells. Time MTS course assays confirmed clone 1, clone 2 and clone 3 Cas9-*MARCH2* HCT116 cells had reduced cell viability compared with control cells (Figure 1b). EdU (5-ethynyl-2’-deoxyuridine) is an alternative to the BrdU assay for directly measuring active DNA synthesis or S phase synthesis during the cell cycle. Clone 1, clone 2 and clone 3 Cas9-*MARCH2* HCT116 cells contained lower percentages of EdU-positive cells (i.e., proliferative cells) than control cells (Figures 1c and d). Colony formation assays demonstrated knockout of *MARCH2* suppressed the colony-forming ability of HCT116 cells (Figures 1e and f). Among the three clones, the most obvious inhibitory effects were observed for clone 3, so this clone was selected for all subsequent experiments.

### Knockout of *MARCH2* promotes apoptosis and cell cycle arrest in the G2/M phase

**Figure 2:**
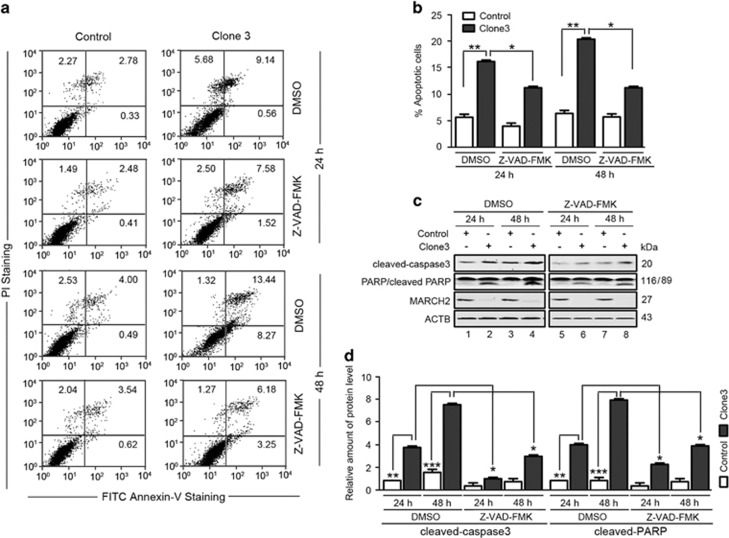
Knockout of *MARCH2* in HCT116 cells promotes apoptosis. (**a**) Control cells and Cas9-*MARCH2* HCT116 cells were serum-starved for 18 h, and then pulsed with 10% FCS for 24 h or 48 h with or without 50 *μ*m z-VAD-fmk. Apoptosis was measured by FITC–Annexin-V/PI staining and flow cytometry. (**b**) Data are mean±S.E.M. of at least three independent experiments. (**c**) Western blotting of the levels of cleaved caspase-3 and cleaved-PARP in Cas9-*MARCH2* HCT116 cells treated as described in (**a**). (**d**) Quantification of cleaved caspase-3 and cleaved-PARP levels relative to ACTB in cells treated as described in (**c**). The average value in DMSO treated control cells for 24 h was normalized to 1. Data are mean±S.D. of three independent experiments. *P<0.05, **P<0.01, ***P<0.001

FITC–Annexin-V and PI staining revealed knockout of *MARCH2* in HCT116 cells increased the number of apoptotic cells, with a time-dependent effect observed (Figures 2a and b). To determine whether knockout of *MARCH2* enhanced apoptosis via the caspase-dependent pathway, Cas9-*MARCH2* HCT116 cells were pretreated with the pan-caspase inhibitor z-VAD-fmk for 2 h, then further cultured for 24 or 48 h. Flow cytometry analysis revealed pretreatment with z-VAD-fmk partly reduced the percentage of apoptotic cells. Western blotting analysis confirmed knockout of *MARCH2* increased the levels of cleaved caspase-3 and PARP, whereas z-VAD-fmk pretreatment attenuated their cleavage in Cas9-*MARCH2* HCT116 cells (Figures 2c and d). These results demonstrate caspase-dependent signaling mediates the increased levels of apoptosis in Cas9-*MARCH2* HCT116 cells.

**Figure 3:**
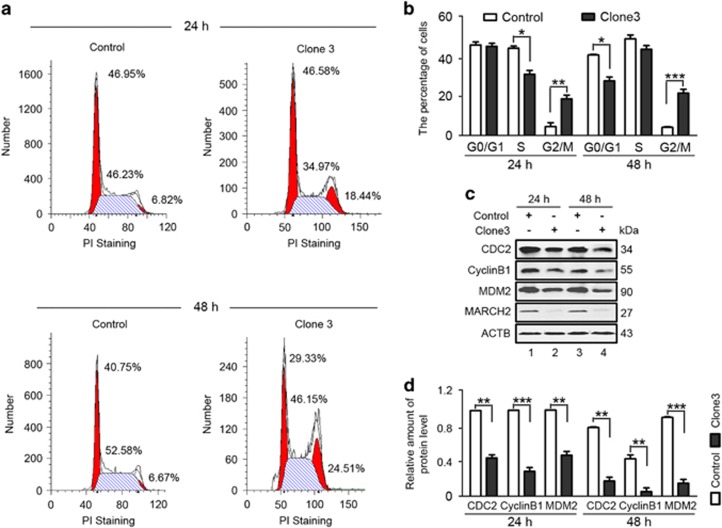
Knockout of *MARCH2* induces G2/M phase arrest. (**a**) Control and Cas9-*MARCH2* HCT116 cells were serum-starved for 18 h, and then pulsed with 10% FBS for 24 h or 48 h. Cell cycle distribution was assayed by flow cytometry. (**b**) Percentages of G0/G1, G2/M and S phase cells. Each bar is mean±S.D. of three independent experiments. (**c**) Western blotting of the levels of CDC2, Cyclin B1 and MDM2 in the indicated cells treated as described in (**a**). (**d**) Quantification of CDC2, Cyclin B1 or MDM2 levels relative to ACTB in cells treated as described in (**c**). Average value for control cells was normalized to 1. Data are mean±S.D. of three independent experiments. *P<0.05, **P<0.01, ***P< 0.001

Based on the preliminary assays in which we determined the effects of MARCH2 KO on cell viability and apoptosis, we explored the effect of MARCH2 inactivation on cell cycle progression by PI staining. As shown in Figures 3a and b, a significantly higher percentage of cells in G2/M-phase were observed in Cas9-*MARCH2* HCT116 cells than in control groups both at 24 h and 48 h. There was a concomitant reduction in the proportion of cells in the S phase at 24 h and G0/G1 phase at 48 h. These data suggest that inhibition of cell growth in Cas9-*MARCH2* HCT116 cells may be associated with the induction of G2/M-phase cell cycle arrest.

The G2/M transition is regulated by the cyclin B1/CDC2 complex^20^ and MDM2 is a negative regulator of p21 involved in the G2/M checkpoint that is required for cell cycle arrest in the G2/M phase.^21, 22^ Further investigation indicated that knockout of *MARCH2* decreased the expression of CDC2, Cyclin B1 and MDM2 (Figures 3c and d). This finding suggested that *MARCH2* KO-induced G2/M phase arrest may be related to the decrease of cyclin B1/CDC2 activities.

### ER stress is associated with autophagy, apoptosis and cell cycle arrest in Cas9-*MARCH2* HCT116 cells

**Figure 4:**
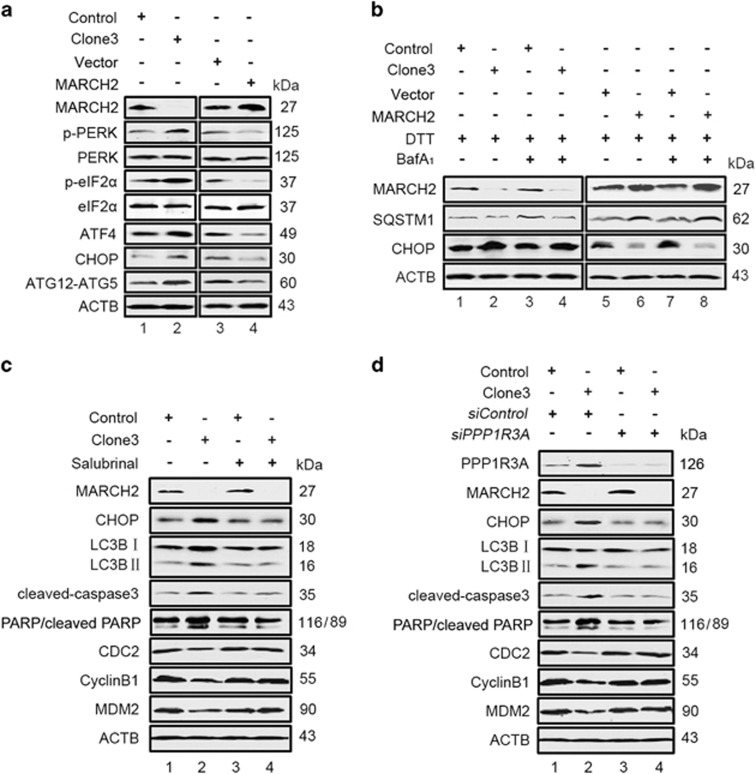
MARCH2 modulates ER stress. (**a**) Western blotting analysis of PERK, p-PERK (Thr 981) and p-EIF2*α* (Ser 51), EIF2*α*, ATF4, CHOP and ATG12-ATG5 levels in the indicated cell lines. ACTB was used as a loading control. (**b**) Control cells and Cas9-*MARCH2* HCT116 cells were treated with BafA1 (10 nM) and/or DTT (1 mM) for 4 h and subjected western blotting to quantify CHOP and SQSTM1. (**c**) Control cells and Cas9-*MARCH2* HCT116 cells were treated with or without salubrinal (5 *μ*M) for 2 h and subjected western blotting to quantify CHOP, LC3B-II, cleaved caspase-3, cleaved-PARP, CDC2, Cyclin B1 and MDM2. (**d**) Cells were transfected with siRNA control or siRNA targeting *PPP1R3A* for 24 h, and subjected Western blotting to quantify endogenous CHOP, LC3B-II, cleaved caspase-3, cleaved-PARP, CDC2, Cyclin B1 and MDM2

As MARCH2 localizes to the ER, we examined whether ER stress exists in Cas9-*MARCH2* HCT116 cells. Western blotting revealed knockout of *MARCH2* in HCT116 cells upregulated UPR molecules including phosphorylated PERK (Thr 981), EIF2*α* (Ser 51) and ATF4 (Figure 4a, lane 2 *versus* lane 1). This effect was accompanied by a significant increase in the amount of ATG12-ATG5 conjugation in Cas9-*MARCH2* HCT116 cells. In contrast, overexpression of MARCH2 decreased the levels of p-PERK, p-EIF2*α*, ATF4, CHOP and ATG12-ATG5 in HCT116 cells (Figure 4a, lane 4 *versus* lane 3). In addition, we also observed the slight increase of cleaved ATF6, p-IRE1*α* (Ser 724) and XBP1s in Cas9-*MARCH2* HCT116 cells (Supplementary Figure 2). Consistent with these findings, levels of the ER stress sensor protein GRP78, Calnexin and calreticulin were upregulated in Cas9-*MARCH2* HCT116 cells (Supplementary Figure 2). These results suggested that *MARCH2* KO-mediated activation of PERK, ATF6 and IRE1*α* pathways differentially contributed to MARCH2-mediated autophagy. Prolonged activation of ER stress often leads to cellular apoptosis that is reflected by increased levels of the protein CHOP. Consistent with our expectation, the increased expression of CHOP was observed in Cas9-*MARCH2* HCT116 cells (Figure 4a, lane 2 *versus* lane 1), thereby implicating appearance of cellular apoptosis in these cells.

To provide further evidence that *MARCH2* KO upregulates ER stress, we investigated the effect of treating HCT116 cells with dithiothreitol (DTT) to induce cellular ER stress. DTT-induced ER stress was confirmed by increased expression of CHOP (Figure 4b, lane 1 and lane 5). In the presence of DTT, knockout of *MARCH2* increased the levels of CHOP and decreased the levels of SQSTM1 (Figure 4b, lane 2 *versus* lane 1, lane 4 *versus* lane 3), indicating loss of *MARCH2* enhanced DTT-mediated ER stress and autophagy. Overexpression of MARCH2 in HCT116 cells downregulated CHOP and increased the accumulation of SQSTM1 (Figure 4b, lane 6 *versus* lane 5, lane 8 *versus* lane 7), suggesting MARCH2 activity impairs DTT-mediated ER stress and autophagy. These findings indicate MARCH2 regulates autophagy, at least in part in association with ER stress signaling.

To further confirm the association with ER stress, Cas9-*MARCH2* HCT116 cells were treated with salubrinal, which acts as a phosphatase inhibitor and increases EIF2*α* phosphorylation leading to global decrease in translation, subsequently lessening ER stress.^23^ As shown in Figure 4c (lane 4 *versus* lane 2), salubrinal reduced the levels of CHOP, accompanied by decreased expression of LC3B-II, cleaved caspase-3 and cleaved-PARP. Moreover, the levels of CDC2, Cyclin B1 and MDM2 were enhanced in the presence of salubrinal (Figure 4c, lane 4 *versus* lane 2). The results implied that the ER stress response is upstream of autophagy, apoptosis and G2/M phase arrest in Cas9-*MARCH2* HCT116 cells.

To further validate the role of ER stress in *MARCH2* KO-mediated induction of autophagy, we employed a genetic approach targeting PPP1R3 A (protein phosphatase 1, regulatory subunit 3A), which is responsible for EIF2*α* dephosphorylation.^24^ As shown in Figure 4d, transfection of *PPP1R3A* siRNA significantly decreased PPP1R3A protein expression at 48 h. Correspondingly, the levels of the downstream protein CHOP were reduced in Cas9-*MARCH2* HCT116 cells transfected with *PPP1R3A* siRNA (Figure 4d, lane 4 *versus* lane 2), implying that *PPP1R3A* silencing inhibited ER stress signaling. Simultaneously, *PPP1R3A* deficiency attenuated the expression of LC3B-II, cleaved caspase-3 and cleaved-PARP, but enhanced the levels of CDC2, Cyclin B1 and MDM2, indicating that knockdown of *PPP1R3A* attenuated autophagy, apoptosis and G2/M phase arrest in Cas9-*MARCH2* HCT116 cells (Figure 4d, lane 4 *versus* lane 2). In view of the fact that the levels of PPP1R3A were increased in Cas9-MARCH2 HCT116 cells (Figure 4d, lane 2 *versus* lane 1), thus implying that increased PPP1R3A may be one of the mechanisms in *MARCH2* KO-induced ER stress.

Next, we analyzed the phenotypes of salubrinal-treated Cas9-*MARCH2* HCT116 cells. Compared with control cells, knockout of *MARCH2* increased GFP-LC3B puncta distribution in the presence of BafA1 alone, whereas salubrinal reversed this effect (Supplementary Figure 3a and b). Flow cytometry analysis revealed salubrinal reversed the increase in apoptosis observed in Cas9-*MARCH2* HCT116 cells (Supplementary Figure 3c and d). Likewise, salubrinal blocked the G2/M phase arrest induced by knockout of *MARCH2* (Supplementary Figure 3e). Taken together, these findings underpin the importance of ER stress responses in the growth arrest induced by knockout of *MARCH2* in HCT116 cells.

### Knockout of *MARCH2* sensitizes HCT116 cells to chemotherapy

**Figure 5:**
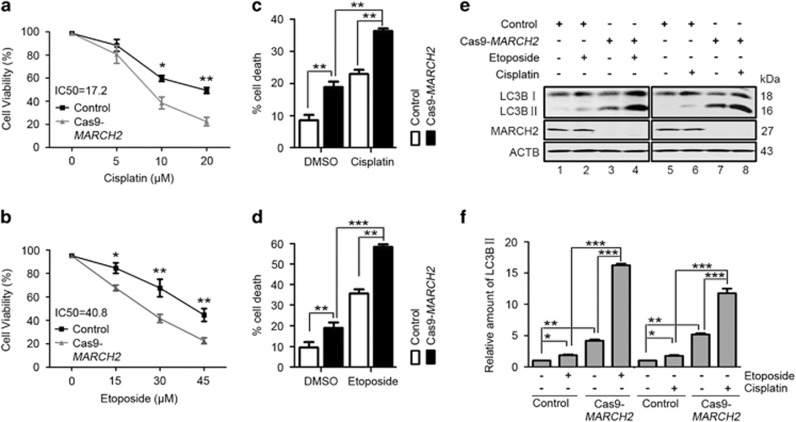
Knockout of *MARCH2* sensitizes HCT116 cells to chemotherapy. (**a, b**) Control cells and Cas9-*MARCH2* cells were cultured in 96-well plates (5000 cells/well, five replicate wells), treated with indicated dose of cisplatin or etoposide for 24 h. Cell viability was assessed using the MTS assay. The IC50 of etoposide or cisplatin is 17.2 *μ*M or 40.8 *μ*M, respectively. Data are mean±S.D. of three independent experiments. (**c, d**) Control cells and Cas9-*MARCH2* HCT116 cells were treated with or without etoposide or cisplatin for 24 h. Apoptosis was quantified by Annexin-V/PI staining and flow cytometry. (**e**) Western blot of the levels of LC3B-II in cells treated as described in (**c**). (**f**) Histogram of the of LC3B-II/ACTB ratio for cells treated as described in **e**. The average value in the control group was normalized to 1. Data are mean±S.D. of three independent experiments. *P<0.05, **P<0.01, ***P<0.001

Given that knockout of *MARCH2* induced growth arrest, we investigated whether knockout of *MARCH2* enhanced the chemosensitivity of colon cancer cells. Etoposide and cisplatin are clinically used for the treatment of several cancers, including colon cancer. Cas9-*MARCH2* HCT116 cells were treated with different concentrations of cisplatin or etoposide for 24 h. MTS assays revealed etoposide and cisplatin reduced the cell viability of HCT116 cells in dose-dependent manners, and these suppressive effects were enhanced in Cas9-*MARCH2* HCT116 cells (Figures 5a and b). Flow cytometry showed that the number of apoptotic cells was higher in cisplatin (10 *μ*M)- or etoposide (30 *μ*M)-treated Cas9-*MARCH2* HCT116 cells than control cells treated with etoposide or cisplatin alone (Figures 5c and d). Western blot analysis showed both etoposide and cisplatin elevated LC3B-II accumulation in control HCT116 cells (Figures 5e and f, lane 2 *versus* lane 1, lane 6 *versus* lane 5). However, etoposide and cisplatin treatment further promoted LC3B-II accumulation in Cas9-*MARCH2* HCT116 cells (Figures 5e and f, lane 4 *versus* lane 2, lane 8 *versus* lane 6). These findings suggest MARCH2 inactivation-mediated autophagy enhanced the sensitivity of colon cancer cells to chemotherapy.

### Overexpression of MARCH2 enhances the tumorigenicity of colon cancer cells *in vivo*

To investigate the influence of overexpressing MARCH2 on colon tumor cell proliferation *in vivo*, three colon cancer cell lines (HCT116, LOVO and HT-29) stably overexpressing MARCH2 were established. MTS assays indicated overexpression of MARCH2 enhanced cell viability (Supplementary Figure 4a and b) and increased the percentage of EdU-positive cells in HCT116 cells, LOVO cells and HT-29 cells (Supplementary Figure 4c and d). These data indicate overexpression of MARCH2 promotes the growth of colon tumor cells. Next we investigated the sensitivity of MARCH2-overexpressing cells to chemotherapeutic drugs. As shown in Supplementary Figure 5a and 5b, both etoposide and cisplatin reduced HCT116 cell viability in a dose-dependent manners, but this suppressive effect could be partially reversed in MARCH2-overexpressing HCT116 cells. The similar results occurred in the detection of apoptosis (Supplementary Figure 5c and 5d), indicating that MARCH2 overexpression decreased the sensitivity of colon cancer cells to chemotherapy.

**Figure 6:**
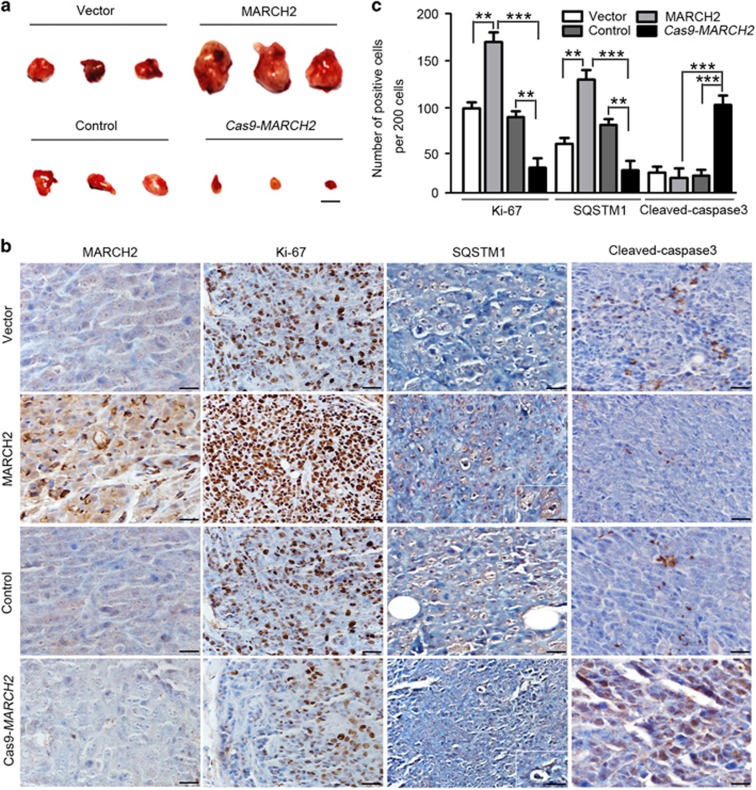
MARCH2 promotes the tumorigenicity of colon cancer cells *in vivo*. (**a**) Empty vector-transfected HCT116 cells, MARCH2-overexpressing HCT116 cells, control (wild-type) HCT116 cells or Cas9-*MARCH2* HCT116 cells were subcutaneously injected into BALB/c nude mice (*n*=6). Xenograft tumors were excised and imaged on day 20. Scale bar: 1 cm. (**b**) Immunohistochemical staining for MARCH2, Ki-67, SQSTM1 and cleaved caspase-3 in xenograft tumor tissues. (**c**) The number of positive cells per 200 cells in (**b**). **P<0.01, ***P<0.001

The *in vivo* effects of MARCH2 were evaluated using a colon cancer xenograft model established in BALB/C nude mice. Mice were subcutaneously injected in the right axilla with empty vector-transfected HCT116 cells, stable MARCH2-overexpressing HCT116 cells, MARCH2 wild-type HCT116 cells or *Cas9-MARCH2* HCT116 cells. At 20 days after inoculation, the mice were sacrificed and the tumors in each mouse were excised and photographed. As shown in Figure 6a, the stable MARCH2-overexpressing group had larger tumors than the vector group, indicating MARCH2 enhanced the tumorigenicity of HCT116 cells. However, the *Cas9-MARCH2* group had smaller tumors than the control group, indicating knockout of *MARCH2* reduced the tumorigenicity of HCT116 cells (Figure 6a).

Next, we examined the expression of MARCH2, Ki-67, SQSTM1 and cleaved caspase-3 in the xenograft tumor sections. Immunohistochemistry revealed MARCH2-overexpressing tumors contained a significantly higher proportion of Ki-67-positive cells than vector control tumors (Figures 6b and c), whereas *Cas9-MARCH2* tumors contained fewer Ki-67-positive cells (Figures 6b and c, lower panel). Simultaneously, SQSTM1 expression was stronger in MARCH2-overexpressing tumors and weaker in *Cas9-MARCH2* tumors than control group. There was no significant difference in cleaved caspase-3 expression between the MARCH2-overexpressing tumors and vector control group; however, cleaved caspase-3 staining was stronger in *Cas9-MARCH2* tumors than vector control tumors (Figures 6b and c, lower panel). Collectively, these data suggest *MARCH2* knockout-mediated autophagy and apoptosis exerted an anti-tumor effect in HCT116 cells *in vivo*.

### Correlation between MARCH2 expression and the clinicopathologic characteristics of colon cancer

**Figure 7:**
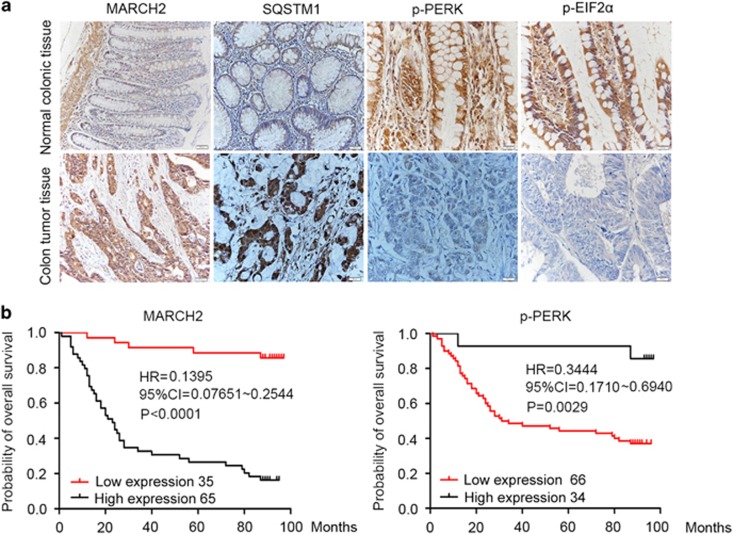
MARCH2 is upregulated in human colon cancer. (**a**) immunohistochemical staining for MARCH2, SQSTM1, p-PERK (Thr 981) and p-EIF2*α* (Ser 51) in human colon cancer tissues and adjacent non-tumor colon tissues. (**b**) Kaplan–Meier survival analysis the correlation between the levels of MARCH2 (or p-PERK) and the survival time in colon cancer patients

We next examined the expression of MARCH2, SQSTM1, p-PERK, p-EIF2*α* and p-IRE1*α* in primary colon cancer tissues and adjacent non-tumor tissues. Weak MARCH2 signals, mainly localized in the cytoplasm, were detected in non-tumor tissues. In contrast, strong or moderate MARCH2 staining was observed in most colon cancer tissues (Figure 7a,Supplementary Figure 6). Similarly to MARCH2, the expression of SQSTM1 was higher in colon cancer tissues than non-tumor tissues. Furthermore, strong or moderate expression of p-PERK (Thr 981) and p-EIF2*α* (Ser 51) were observed in the non-tumor tissues adjacent to cancer, with weak or absent signals in colon cancer tissues. There was no significant difference in the expression of p-IRE1*α* between colon cancer tissues and the non-tumor tissues adjacent to cancer (Supplementary Figure 7). These data further imply that high expression of MARCH2 is accompanied by decreased PERK/EIF2*α* signaling and autophagy.

We next analyzed the relationship between MARCH2 expression and the clinicopathologic characteristics of 100 patients with colon cancer (Table 1). High MARCH2 expression was significantly associated with larger tumor size (odds ratio (OR)=12.27, *P*<0.001), histological grade (OR=6.58, *P* =0.004), interstitial invasive depth (OR=23.33, *P<0.001*), advanced tumor stage (OR=5.22, *P* =0.012) and positive lymphatic metastasis (OR=11.84, *P*<0.001). These data suggest high level MARCH2 expression is positive associated with malignant potential in colon cancer.

We also assessed the association between MARCH2 expression and survival; 100 patients with colon cancer were followed-up for up to 98 months. During the observation period, 60 (60%) patients died of recurrence. The 8-year cumulative survival rate for all patients was 40%. Patients with moderate to high expression of MARCH2 (++ to +++ Supplementary Figure 6) had poorer overall survival compared with patients with negative to low expression of MARCH2 (− to + Figure 7b). In contrast, patients with negative to low expression of p-PERK had poorer overall survival compared with patients with moderate to high expression of p-PERK. Furthermore, correlation analysis demonstrated MARCH2 expression correlated negatively with the levels of p-PERK in colon carcinoma (Table 2 and Supplementary Figure 6).

## Discussion

In this study, we investigated the function and mechanism of action of MARCH2 during the development of colon cancer *in vitro* and *in vivo*. Overexpression of MARCH2 promoted the proliferation of HCT116 cells, whereas knockout of *MARCH2* suppressed cell viability and promoted apoptosis and G2/M phase cell cycle arrest. Further investigation revealed these effects were closely associated with ER stress in *MARCH2* knockout cells, via a mechanism involving activation of PERK-EIF2*α*-ATF4-CHOP signaling. Importantly, knockout of *MARCH2* increased the sensitivity of colon cancer cells to etoposide or cisplatin. Moreover, high MARCH2 expression was associated with poorer overall survival in colon cancer, indicating that expression of MARCH2 may represent an independent prognostic factor for overall survival. In addition, overexpression of MARCH2 was accompanied by decreased autophagy, as indicated by reduced LC3B expression and enhanced SQSTM1 expression, and decreased ER stress signaling, as reflected by reduced PERK and ATF6 levels. This is the first evidence of a relationship between MARCH2, ER stress and autophagy in colon carcinoma, and indicates inactivation of MARCH2 may represent a novel therapeutic target for colon cancer.

The ER stress pathway has an important role in the development of cancer.^25^ High level activation of UPR molecules is observed in a wide range of primary human tumors, including glioblastoma and carcinomas of the breast, stomach, esophagus, and liver.^26, 27, 28, 29, 30, 31^ In contrast, somatic mutations in *IRE1α* or *PERK*, in most cases loss-of-function mutations, are rarely (<1%) detected in these tumors.^32, 33, 34^ Despite the overwhelming evidence for ongoing ER stress and UPR activation in many forms of cancer, whether this ultimately inhibits or promotes tumor growth remains an area of intense debate.^8, 26, 35^ Most evidence arguing that the UPR supports tumor growth comes from xenograft studies in mice, in which genetic deletion of one or more branches of the UPR or altering the expression of ER chaperones reduces the growth of tumor cells *in vivo*. Xie *et al.*^36^ reported that mild ER stress through BiP-PERK- EIF2*α* pathway has a critical role in protecting against RIBE-induced cellular damage and hence may potentially decrease the risk of secondary cancer after radiotherapy. Pytel *et al.*^37^ showed PERK is a haploinsufficient tumor suppressor, as the gene dose determines the tumor-suppressive *versus* tumor-promoting properties of PERK in melanoma. Here, we proved inactivation of *MARCH2* in HCT116 cells increased GRP78/BIP, subsequent PERK/EIF2*α* signaling and the levels of downstream CHOP. The immunohistochemistry results suggested that MARCH2 expression correlated negatively with the levels of p-PERK in colon cancer. These observations suggest that *MARCH2* KO-mediated PERK/EIF2*α* pathway may contribute to tumor suppression. How *MARCH2* KO affects ER stress remains unclear, which deserves further investigation in the future. As an E3 ligase localized in the ER, MARCH2 may maintain the ER homeostasis under normal circumstances via unknown mechanism. Under MARCH2 inactivation, such homeostasis maybe had broken, which caused the accumulation of unfolded or misfolded proteins in the lumen of the ER. Molecular chaperones such as GRP78/BIP dissociated from the UPR sensors, leading to their activation. We will further explore this hypothesis. Similarly, the relationship between MARCH2 and ER stress in colon carcinoma also needs further investigation.

Elevated SQSTM1 is considered a hallmark for impaired autophagy and has been associated with poor prognosis in some tumor types. Burdelski *et al.*^38^ found high levels of SQSTM1 were significantly associated with increased tumor cell proliferation in prostate cancer. New evidence supports a key autophagy-independent role for the adaptor SQSTM1 in the signaling functions central to tumor initiation in the epithelium and suppression of tumor progression in the stroma.^39^ Schläfli *et al.*^40^ reported the autophagy markers LC3 and SQSTM1 had prognostic value in early-stage non-small cell lung cancer. In this study, high MARCH2 expression was strongly associated with the clinicopathological features of aggressive disease, including larger tumor size, higher histological grade, deeper interstitial invasion, advanced tumor stage and positive lymph node metastasis. Furthermore, high MARCH2 expression correlated with increased levels of SQSTM1, supporting the hypothesis that MARCH2-regulated autophagy has an important role in the development of colon cancer. Therefore, SQSTM1 and MARCH2 may represent prognostic biomarkers of malignant change in colon cancer. Moreover, as knockout of *MARCH2* sensitized colon tumor cells to etoposide or cisplatin treatment, this approach merits further exploration as a therapeutic strategy for colon cancer.

## Materials and methods

### Antibodies and reagents

The following antibodies were used: Rabbit anti-mouse p62/SQSTM1 antibody (MBL International, Woburn, MA, USA; PM045), anti-LC3B (Sigma Aldrich, St. Louis, MO, USA; L7543), anti-Ki-67 (Pierce, Appleton, WI, USA, PA516446), anti-ATG5 (Cell Signaling Technology, Danvers, MA, USA, 12994 S), anti-CHOP (Cell Signaling Technology, 2895 P), anti-GRP78 (Bioss, Scotland, UK, bs-1219 R), anti-PERK (Abcam, Cambridge, UK, ab65142), anti-Phospho-PERK (Santa Cruz Biotechnology, California, CA, USA, sc-32577), anti-EIF2*α* (Cell Signaling Technology, 5324 P), anti-Phospho-EIF2*α* (Cell Signaling Technology, 3398 S), anti-IRE1*α* (Abcam, AB124945), anti-XBP1s (Biolegend, California, CA, USA, 647501), anti-ATF6*α*(F-7) (Santa Cruz Biotechnology, sc166659), monoclonal anti-ATF6 antibody (Imagnex, Littleton, CO, USA, IMG-273), anti-Calnexin (AF-18) (Abcam, ab31290), anti-Calreticulin (Abcam, ab4), anti-ATF4 (Cell Signaling Technology, 11815 S), anti-CDC2 (Santa Cruz Biotechnology, sc-54), anti-MDM2 (Abcam, ab137413), anti-cyclin B1 (Santa Cruz Biotechnology, sc-245), anti-cleaved-PARP (Cell Signaling Technology, 5625 S), anti-cleaved caspase-3 (Abcam, ab52293), anti-PPP1R3A (Abcam, ab104986). Secondary antibodies included DyLight 800/DyLight 680-conjugated IgG against mouse (Rockland, Philadelphia, PA, USA, 610-145-002/610-144-002) or rabbit (Rockland, 611-145-002/611-144-002). Other reagents used in this study were: Bafilomycin A_1_ (BafA_1,_ Sigma Aldrich, B1793), DTT (DTT, Merck, Kenilworth, NJ, USA, 233155), 3-MA (Sigma Aldrich, M9281), Z-VAD-FMK (Santa Cruz Biotechnology, sc-311561), Hoechst 33342 (Sigma Aldrich, 14533).

### Cell culture, transfection and treatments

HCT116, LOVO, HT-29 cells were cultured in DMEM (12800-017; Invitrogen, Carlsbad, CA, USA) supplemented with 10% fetal bovine serum and maintained at 37 °C in a humidified incubator with 5% CO_2_. Cells were transfected with pcDB-MARCH2 or empty vector plasmids using MegaTran 1.0 Transfection Reagent (TT200004; ORIGEN, Rockville, MD, USA) according to the manufacturer’s instructions, and selected using G418 to establish MARCH2-overexpressing cell lines; empty vector-transfected cells were generated as a control.

### Knockout of *MARCH2* using CRISPR/Cas9-mediated genome editing

A MARCH2 knockout HCT166 cell line was established by CRISPR/Cas9-mediated genome editing. The target sequences for CRISPR interference were designed by Shanghai Biomodel Organism Science & Technology Development Co., Ltd., Shanghai, China. The target sequence for human *MARCH2* was TCCAAGGTCGTGGAGGCTACGGG (Exon 2). The clones were screened by sequencing.

### Cell viability assay

Cell viability assays were performed using the CellTiter 96 AQueous One Solution Cell Proliferation Assay (G3582; Promega, Madison, WI, USA) according to the manufacturer’s protocols. Cell viability was calculated as follows: cell viability=absorbance of test group/absorbance of control cell group × 100%. Each experiment was performed in three replicate wells and independently repeated three times.

### EdU incorporation assay

Cell proliferation was detected using the EdU detection kit (C10639; Invitrogen) according to the manufacturer’s protocol. Briefly, cells were plated on coverslips and EdU was added to the medium 4 h before harvesting cells. Cells were fixed in 4% paraformaldehyde and nuclei were counterstained with Hoechst 33342.

### Colony formation assay

The control cells (wild-type) and Cas9-*MARCH2* HCT116 cells were cultured in 24-well plate with 200 cells/well and in five replicate wells. After 2 weeks, colonies with ≥50 cells were counted after crystal violet staining.

### Flow cytometric analysis

To assess the cell cycle, cells were seeded into six-well plates, incubated for different times, collected, fixed in 70% ice-cold ethanol overnight, incubated in RNase (100* μ*g/ml) for 30 min, stained with PI (50* μ*g/ml) and analyzed using a FACSCalibur flow cytometer (Becton Dickinson, Franklinlake, NJ, USA). During the analysis of cell cycle, the death cells and cell debris and aggregates were excluded. We only analyzed ‘gated’ cells.

Apoptosis was detected using the FITC–Annexin-V/PI staining detection kit (Beijing Biosea Biotechnology Co., Ltd., Beijing, China) according to the manufacturer’s protocol. Cells were analyzed on a FACSCalibur flow cytometer (Becton Dickinson).

### Western blot

Total cellular proteins were extracted using radio immunoprecipitation assay buffer (50 mM Tris, pH 7.4, 150 mM NaCl, 1% Triton X-100, 1% sodium deoxycholate, 0.1% SDS, with freshly-added proteinase inhibitor cocktail and phosphatase inhibitors). Equal amounts of proteins were separated by SDS-PAGE and transferred to nitrocellulose membranes, then membranes were blocked and incubated with the indicated antibodies. Blots were visualized using an IRDye 800CW-conjugated or Alexa Fluor 680 secondary antibody, and imaged using an Odyssey infrared imaging system (LI-COR Biosciences, Lincoln, NE, USA).

### Immunofluorescence, fluorescence and confocal microscopy

Cells were cultured in confocal dishes and treated as indicated, fixed with 4% paraformaldehyde and permeabilized with 0.2% Triton X-100 (ST795; Beyotime, Shanghai, China). The dishes were then incubated with FBS overnight and exposed to primary antibody for 1 h at 4 °C. After washing three times with phosphate-buffered saline, cells were incubated with FITC-conjugated secondary antibody and nuclei were stained with Hoechst 33342. Morphological alterations were imaged using an Olympus FluoViewTM FV1000 Confocal Microscope (Olympus, Melville, NY, USA). The number of GFP-LC3B puncta per cell was assessed in 50 cells, and statistical data were obtained from three independent experiments.

### Tumorigenicity assay in nude mice

A nude mouse xenograft model was established using 6–8 week-old female BALB/c nude mice (Experimental Animal Center, Peking University Health Sciences Center, Beijing, China). Mice were housed and maintained in a pathogen-free facility, and all experimental procedures and protocols were approved by the Institutional Authority for Laboratory Animal Care of Peking University. Empty vector HCT116 cells, MARCH2-overexpressing HCT116 cells, control (wild-type) HCT116 cells or Cas9-*MARCH2* HCT116 cells were subcutaneously injected in the right axilla of BALB/c nude mice (*n*=6) in a total volume of 100 *μ*l (4 × 10^6^ cells).^41^ At 20 days after inoculation, the mice were humanly killed; and the tumors were excised and photographed. Tumor tissues were subjected to immunohistochemistry with anti-MARCH2, anti-Ki-67, anti-SQSTM1 and anti-caspase-3 antibodies.

### Colon carcinoma specimens, tissue microarray and immunohistochemical staining

For the retrospective study, archived paraffin-embedded pathologic specimens from 15 patients with primary colon carcinoma were obtained from the archives of the Affiliated Hospital of Shandong Medical College. Archived formalin-fixed, paraffin-embedded non-tumor tissues, located >2 cm away from the paraffin-embedded pathologic specimens were also collected. This study was approved by the medical ethics committee of our institutes.

Colon adenocarcinoma tissue microarray (TMA, Lot No: XT15-035) was obtained from Shanghai Outdo BioTech CO., Ltd., Shanghai, China. The clinicopathologic characteristics and follow-up information for the 100 cases of colon adenocarcinoma are shown in Supplementary Table 1. The colon carcinoma specimens and TMA were processed for immunohistochemical staining. In brief, slides were incubated with the indicated primary antibodies overnight at 4 °C, followed by EnVision/HRP (DAKO, Glostrup, Denmark) for 1 h at room temperature, then peroxidase reactivity was visualized using DAB substrate Kit (DAKO). Finally, sections were counterstained with hematoxylin. Expression of the indicated proteins was quantified using the immunoreactive score (IRS). Staining intensity was graded as 0 (negative), 1 (weak), 2 (moderate) or 3 (strong), and the percentage of positive tumor cells was graded as 0 (≤5%), 1 (6~25%), 2 (26~50%), 3 (51~75%) or 4 (≥76%). The IRS was calculated as the product of scores for staining intensity and percentage of positive cells, and classified as 0–1 (–, negative), 2–4 (+, weak staining), 5–8 (++, moderate staining), and above 9 (+++, strong staining). Those scores of ‘−‘ and ‘+’ were characterized as low expression of protein, whereas scores of ‘++’ and ‘+++’ were recognized as high expression of protein.

### Statistical analysis

SPSS 15.0 software (Chicago, IL, USA) was used for statistical analysis. The *χ*^2^-test was employed for comparisons between groups, and correlations between two variables were evaluated using the Spearman’s rank correlation test. *P*<0.05 was considered statistically significant.

## Figures and Tables

**Table 1:** MARCH2 expression and clinicopathologic features of 100 cases of colon cancer

**Clinicopathologic feature**	***n***	**MARCH2 expression (%)**	***OR*****-value**	***P*****-value**
*Gender*				
Male	55	47 (85.5)	1.11	>0.05
Female	45	39 (86.7)		
				
*Age (years)*				
<60	31	27 (87.1)	0.87	>0.05
≥60	69	59 (85.5)		
				
*Tumor diameter*				
<4cm	20	11 (35.0)	12.27	<0.001
≥4cm	80	75 (98.8)		
				
*Degree of differentiation*				
Well~moderate	53	41 (77.3)	6.58	0.004
Poor	47	45 (95.7)		
				
*Depth of invasion*				
T1~T2	7	2 (28.6)	23.33	<0.001
T3~T4	93	84 (90.3)		
				
*Lymph node metastasis*				
No	58	45 (77.6)	11.84	<0.001
Yes	42	41 (97.6)		
				
*TNM stage*				
I–II	58	46 (79.3)	5.22	0.012
III–IV	42	40 (95.2)		

**Table 2:** Correlation between MARCH2 expression and p-PERK expression in colon cancer tissues

	**MARCH2 expression**		***P*****-value**
	**Negative (*****n***)	**Positive (*****n***)	
*p-PERK expression*			
Negative (*n*)	4	62	<0.01
Positive (*n*)	10	24	
