# Supplementary material for: Knockout of MARCH2 inhibits the growth of HCT116 colon cancer cells by inducing endoplasmic reticulum stress
Source: Cell Death Dis. 2017 Jul 27;8(7):e2957–. doi: 10.1038/cddis.2017.347 (PMC5584615; doi:10.1038/cddis.2017.347)
Supplement: Supplementary Information [file cddis2017347x1.docx]

**Supplementary data**

**Knockout of *MARCH2*** [**inhibits the growth of HCT116 colon cancer cells by inducing endoplasmic reticulum stress**](http://www.ncbi.nlm.nih.gov/pubmed/26623729)

Dan Xia^1,2,3^, Wanli Ji^4^, Kunxiao Wang^1,3^, Chentong Xu^1,3^, Xin Lin^1,3^, Yan Xia^1,3^, Ping Lv^1,3^, Quansheng Song^1,3^, Dalong Ma^1,3^, Yingyu Chen^1,3,^*

^1^ Department of Immunology, Peking University School of Basic medical science; Key Laboratory of Medical Immunology, Ministry of Health, Peking University Health Sciences Center, 38 Xueyuan Road, Beijing, 100191, China.

^2^ Department of Pathology; Shandong Medical College; Jucai Six Road, Linyi, 276000, China.

^3^ Center for Human Disease Genomics, Peking University, 38 Xueyuan Road, Beijing, 100191, China.

^4^ Department of Pathology; Affiliated Hospital of Shandong Medical College; 80 Jintan Road, Linyi, 276000, China.

*Corresponding author: Yingyu Chen, Department of Immunology, Peking University School of Basic medical science, 38 Xueyuan Road, Beijing, 100191, China. Tel & Fax: +86-10-82801149; E-mail: [yingyu_chen@bjmu.edu.cn](mailto:yingyu_chen@bjmu.edu.cn)

**Supplementary Figure Legends**

**Supplementary Figure 1. Generation of *MARCH2* knockout cell lines by CRISPR/Cas9-mediated genome editing*.*** (**a-c**) Sequencing diagrams for Cas9-*MARCH2* knockout clone 1, clone 2 and clone 3.

**Supplementary Figure 2. MARCH2 modulates autophagy through ER stress pathway.** Western blotting analysis of endogenous GRP78/BiP, CRT, CNX, cleaved ATF6, full ATF6, p-IRE1a (Ser724), XBP1s and ATG12-ATG5 in the indicated cell lines. ACTB was employed as a loading control.

**Supplementary Figure 3. Salubrinal attenuates autophagy, apoptosis and cell cycle arrest in Cas9-*MARCH2* HCT116 cells.** (**a**) Representative confocal microscopy images of GFP-LC3B distribution in control cells or Cas9-*MARCH2* HCT116 cells transfected with the GFP-LC3B plasmid for 48 h, then treated with BafA1 (10 nM) for the last 4 h and/or salubrinal (5 μM) for 2 h. Scale bar: 25 mm. (**b**) Quantification of the numbers of GFP-LC3B puncta per cell for cells treated as described in (**a**). Data are mean ± SD of at least 50 cells scored. (**c**) Representative apoptotic profiles. Control (wild-type) cells and Cas9-*MARCH2* HCT116 cells were serum-starved for 18 h, then pulsed with 10% FCS for 48 h in 5 μm salubrinal for 2 h and subjected to PI/Annexin-V double staining and flow cytometry. (**d**) Percentages of apoptotic cells [Annexin-V Positive (%) + PI Positive (%)]. Data are mean ± SEM of at least three independent experiments.. (**e**) Cell cycle distribution of control (wild-type) cells and clone 3 Cas9-*MARCH2* HCT116 cells. Cells were serum-starved for 18 h, pulsed with 10% FCS for 48 h and subjected to flow cytometry. ***P* < 0.01

**Supplementary Figure 4. Overexpression of MARCH2 promotes** **colon cancer cell growth.** **(a)** Western blotting of MARCH2 in colon cancer cell lines. ACTB was used as the loading control. **(b)** Vector or MARCH2-overexpressing HCT116, LOVO and HT-29 cell lines were cultured in 96-well plates (3000 cells/well, five replicate wells), serum-starved for 18 h, pulsed with 10% FCS for 1-7 days, then cell viability was assessed using MTS assay. The average values of vector-transfected cells at 24 h were normalized to 1. Data are mean ± SD of three independent experiments. **(c)** Control and MARCH2-overexpressing HCT116, LOVO and HT-29 cell lines were plated in glass slides in 24-well plates (1000 cells/well; three replicates), serum-starved for 18 h, pulsed with 10% FCS for 48 h, followed by EdU incorporation for 4 h. Nuclei were stained with Hoechst 33342. **(d)** Quantification of the percentage of EdU positive cells (in 200 cells) in from five randomly selected fields for view in each slide. Each bar is mean ± SD of three independent experiments. **P* < 0.05, ***P* < 0.01.

**Supplementary Figure 5. MARCH2 overexpression attenuates the sensitivity of cisplatin and etoposide.** (a and b) Empty vector-transfected HCT116 cells and MARCH2-overexpressing HCT116 cells were cultured in 96-well plates (5000 cells/well, five replicate wells), treated with indicated dose of cisplatin or etoposide for 24 h. Cell viability was assessed using the MTS assay. Data are mean ± SD of three independent experiments. (c and d) Empty vector-transfected HCT116 cells and MARCH2-overexpressing HCT116 cells were treated with or without cisplatin (10 μM) or etoposide (30 μM) for 24 h. Apoptosis was quantified by FITC-Annexin-V/PI staining and flow cytometry. **P* < 0.05.

**Supplementary Figure 6. Immunohistochemical staining for MARCH2 and p-PERK in human colon cancer tissues.** Expression of MARCH2 or p-PERK (Thr 981) was detected by TMA immunohistochemical staining (negative -, low +, moderate ++, high +++). Those scores of “-” and “+” were characterized as low expression of MARCH2 or p-PERK, whereas scores of “++” and “+++” were recognized as high expression of MARCH2 or p-PERK.

**Supplementary Figure 7. Immunohistochemical staining for p-IRE1α (Ser724) in human colon cancer tissues and adjacent non-tumor colon tissues.**
