# Supplementary figures and images for: Knockout of MARCH2 inhibits the growth of HCT116 colon cancer cells by inducing endoplasmic reticulum stress
Source: Cell Death Dis. 2017 Jul 27;8(7):e2957–. doi: 10.1038/cddis.2017.347 (PMC5584615; doi:10.1038/cddis.2017.347)

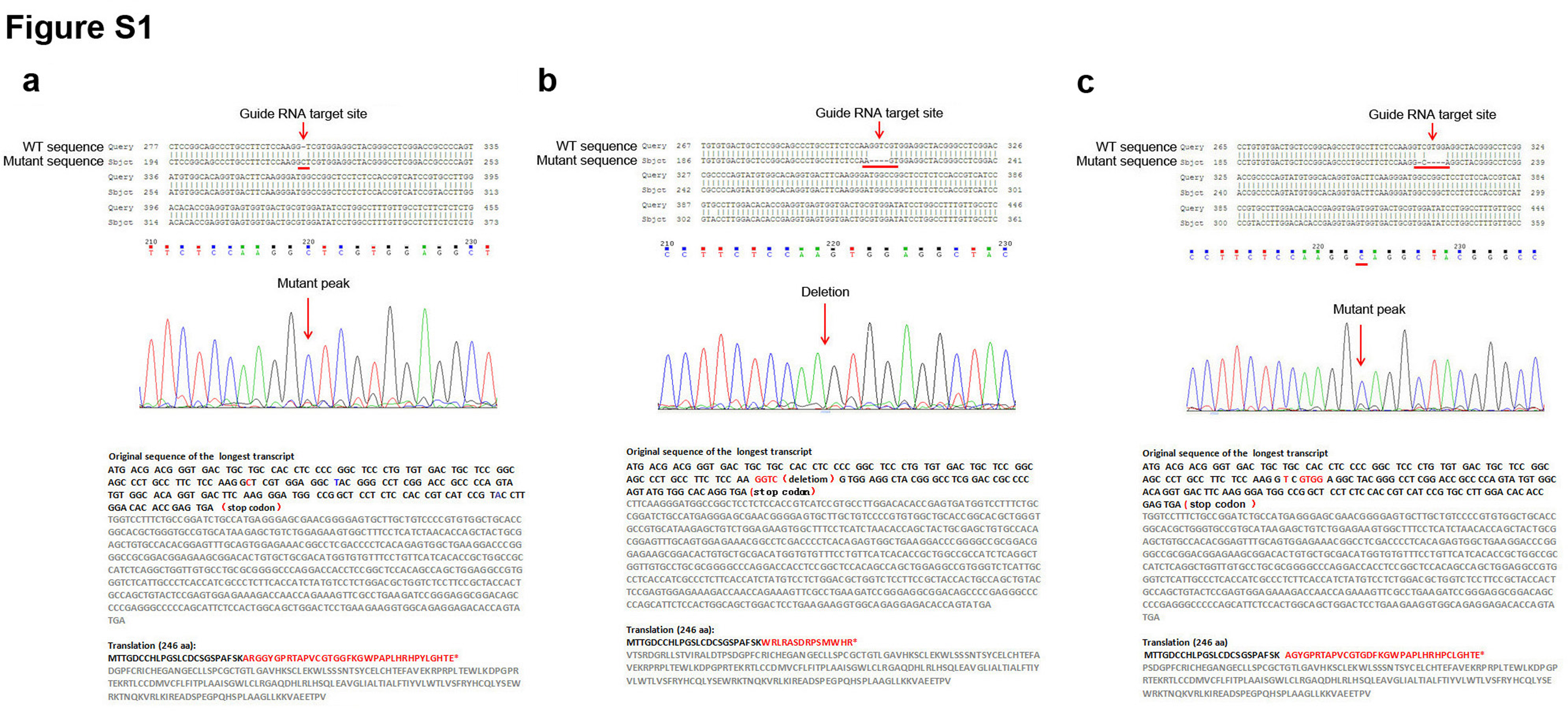

Supplement: Supplementary Figure 1 [file cddis2017347x2.tif]

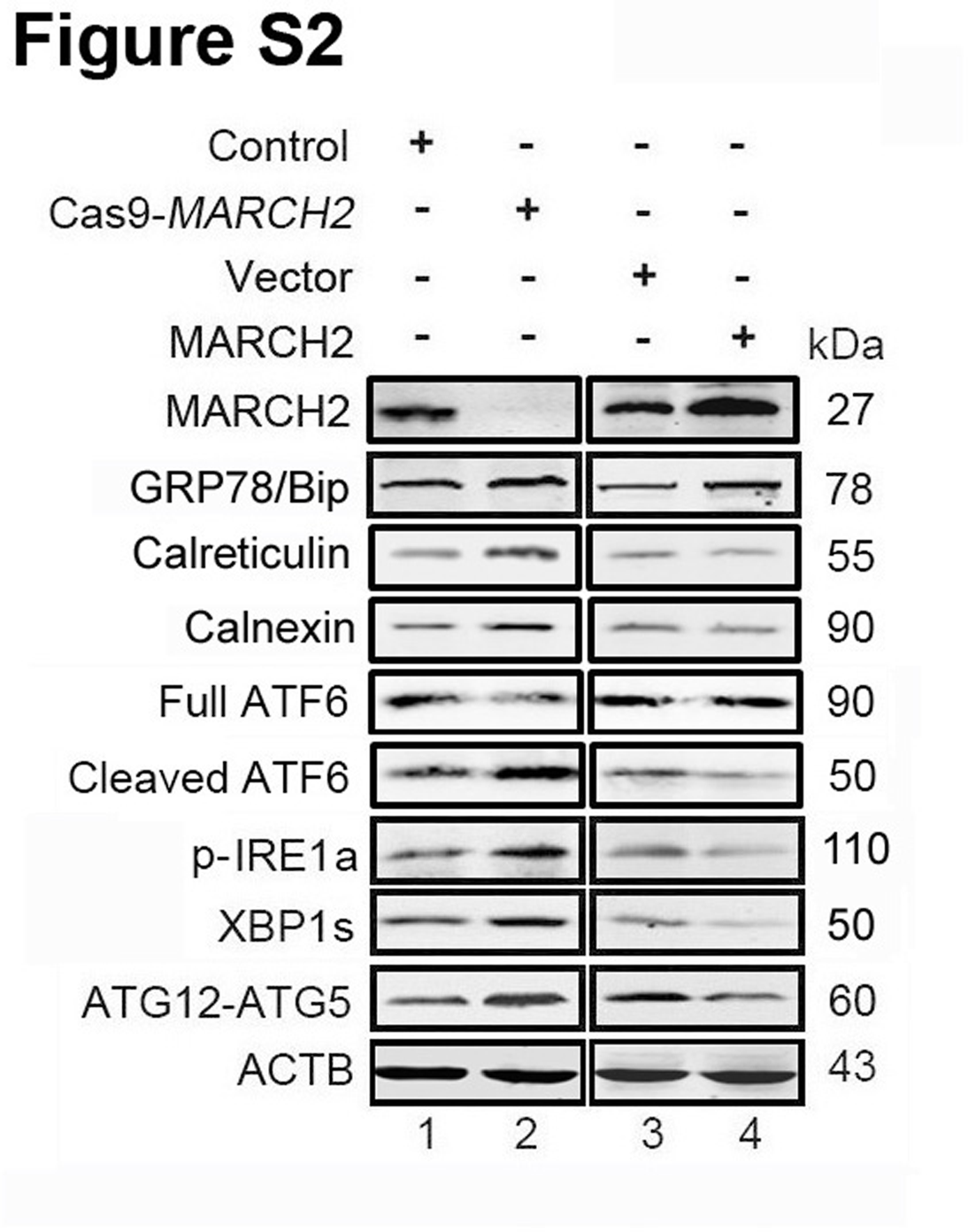

Supplement: Supplementary Figure 2 [file cddis2017347x3.tif]

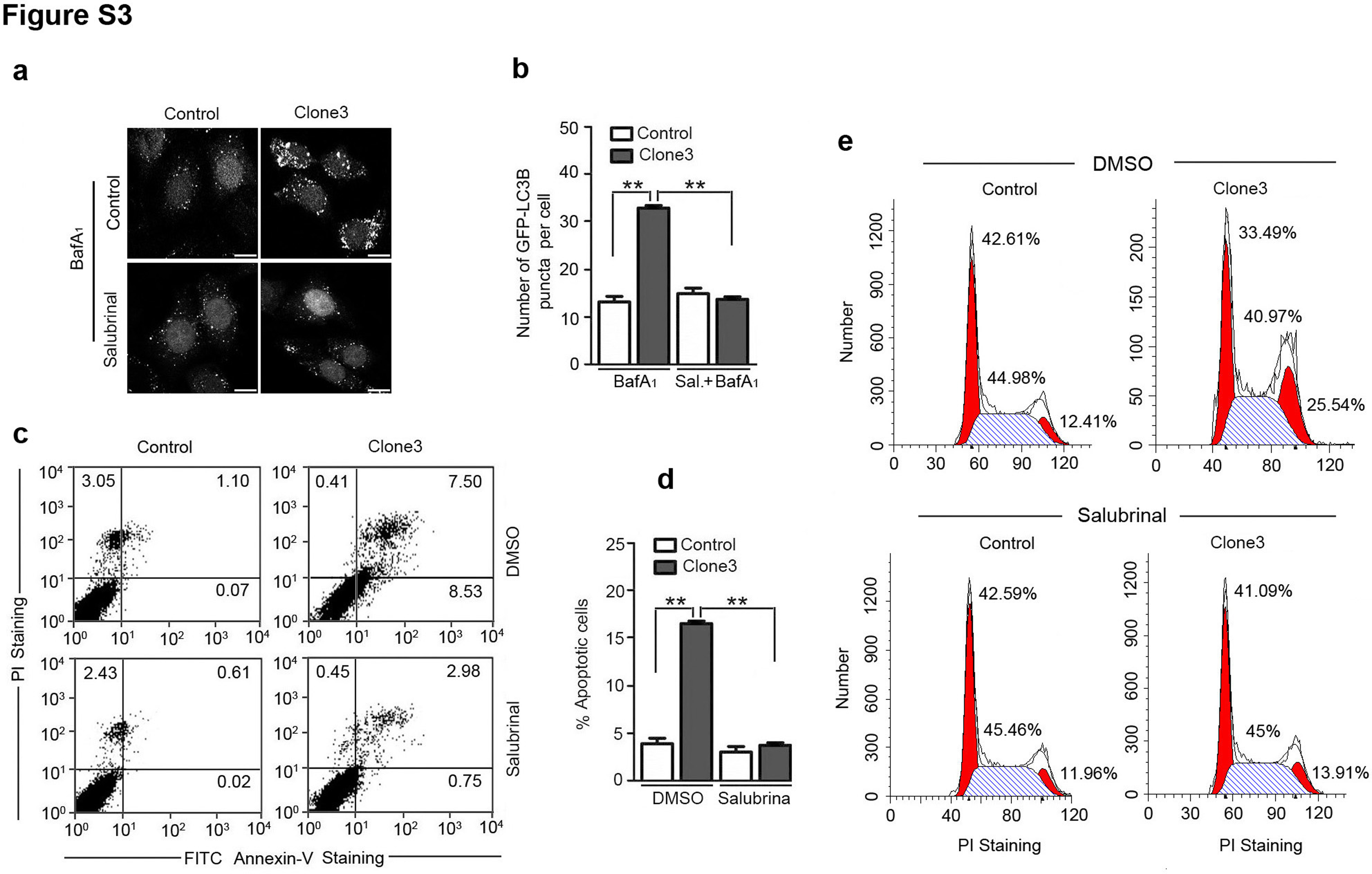

Supplement: Supplementary Figure 3 [file cddis2017347x4.tif]

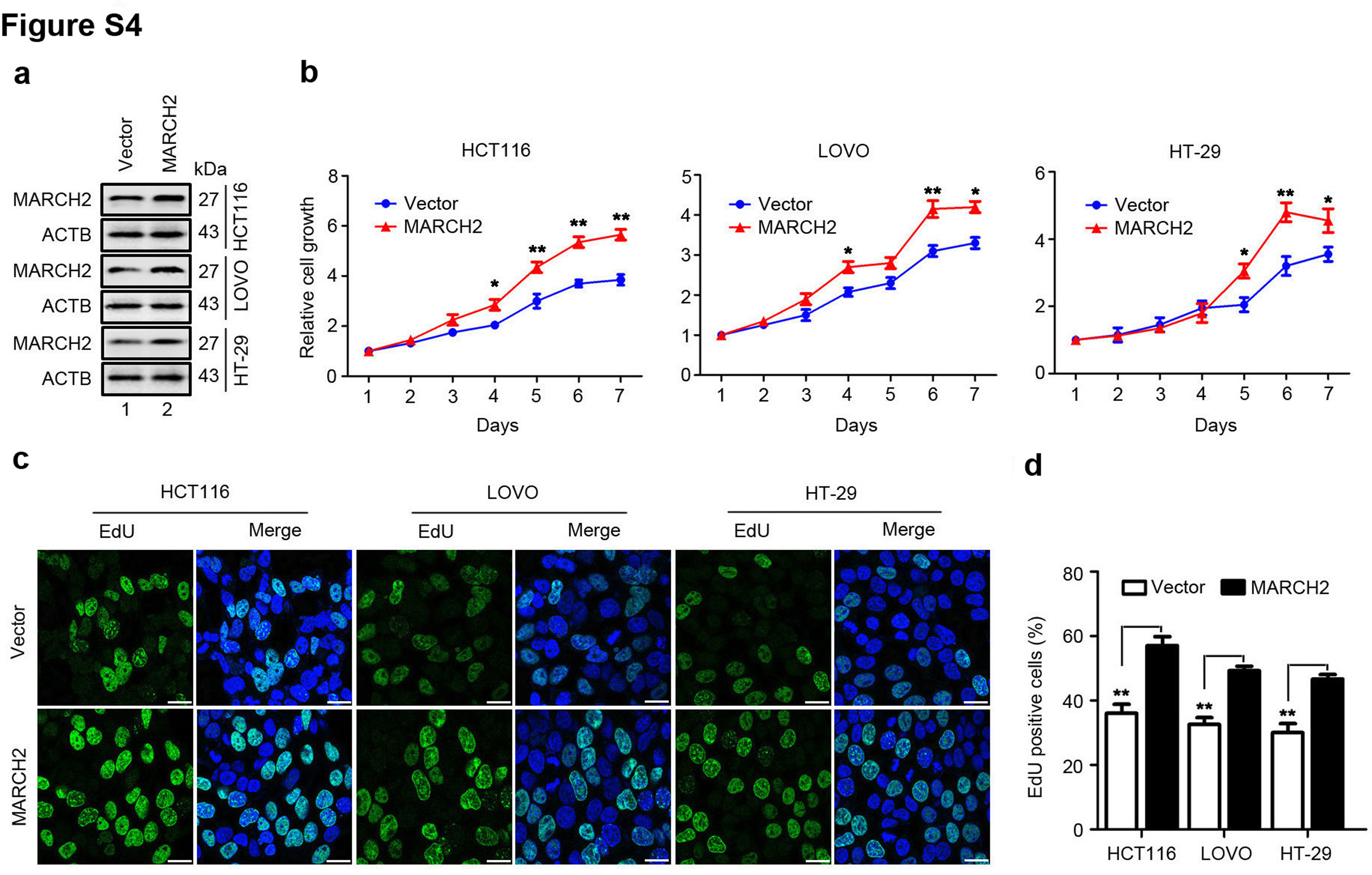

Supplement: Supplementary Figure 4 [file cddis2017347x5.tif]

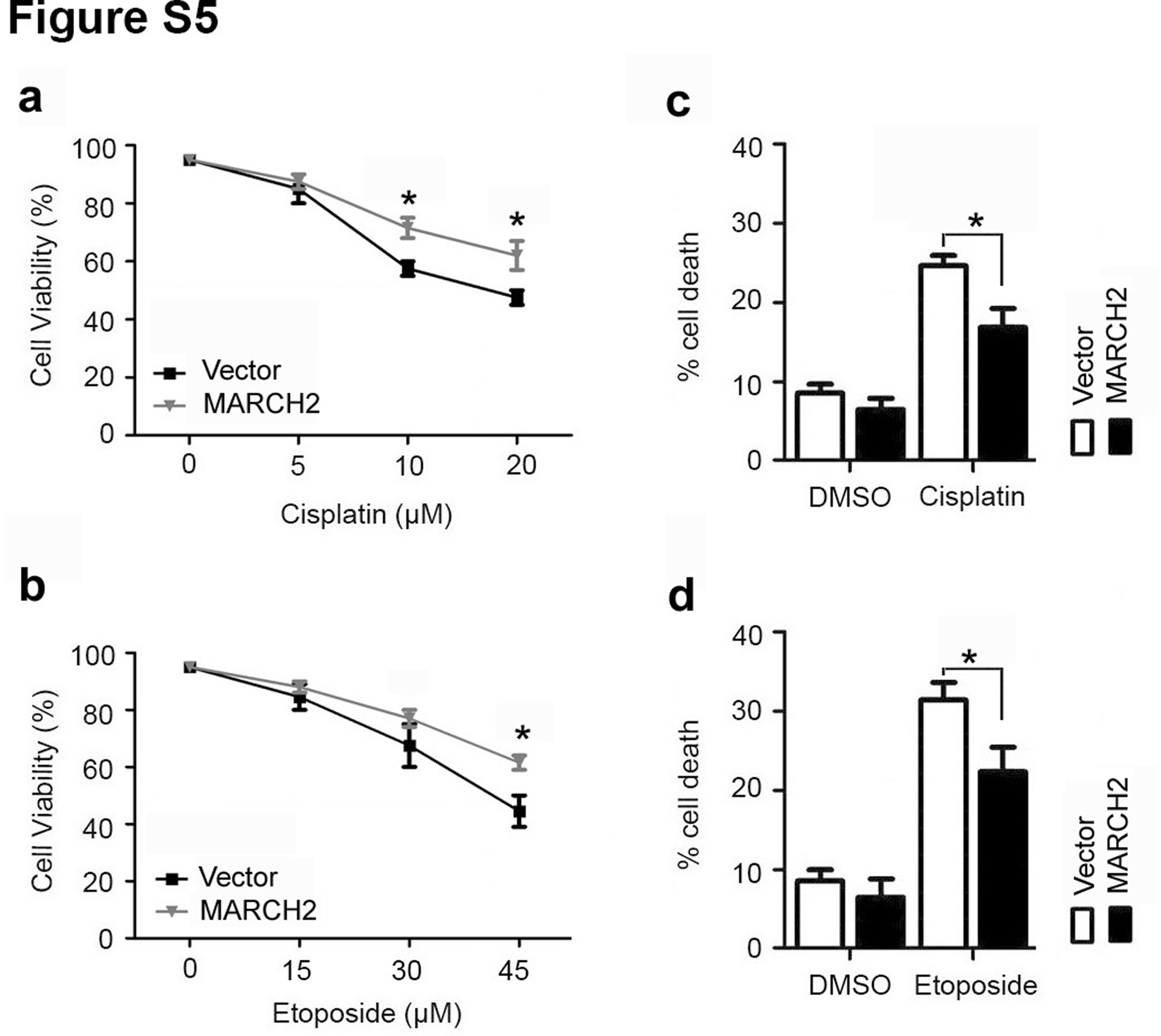

Supplement: Supplementary Figure 5 [file cddis2017347x6.tif]

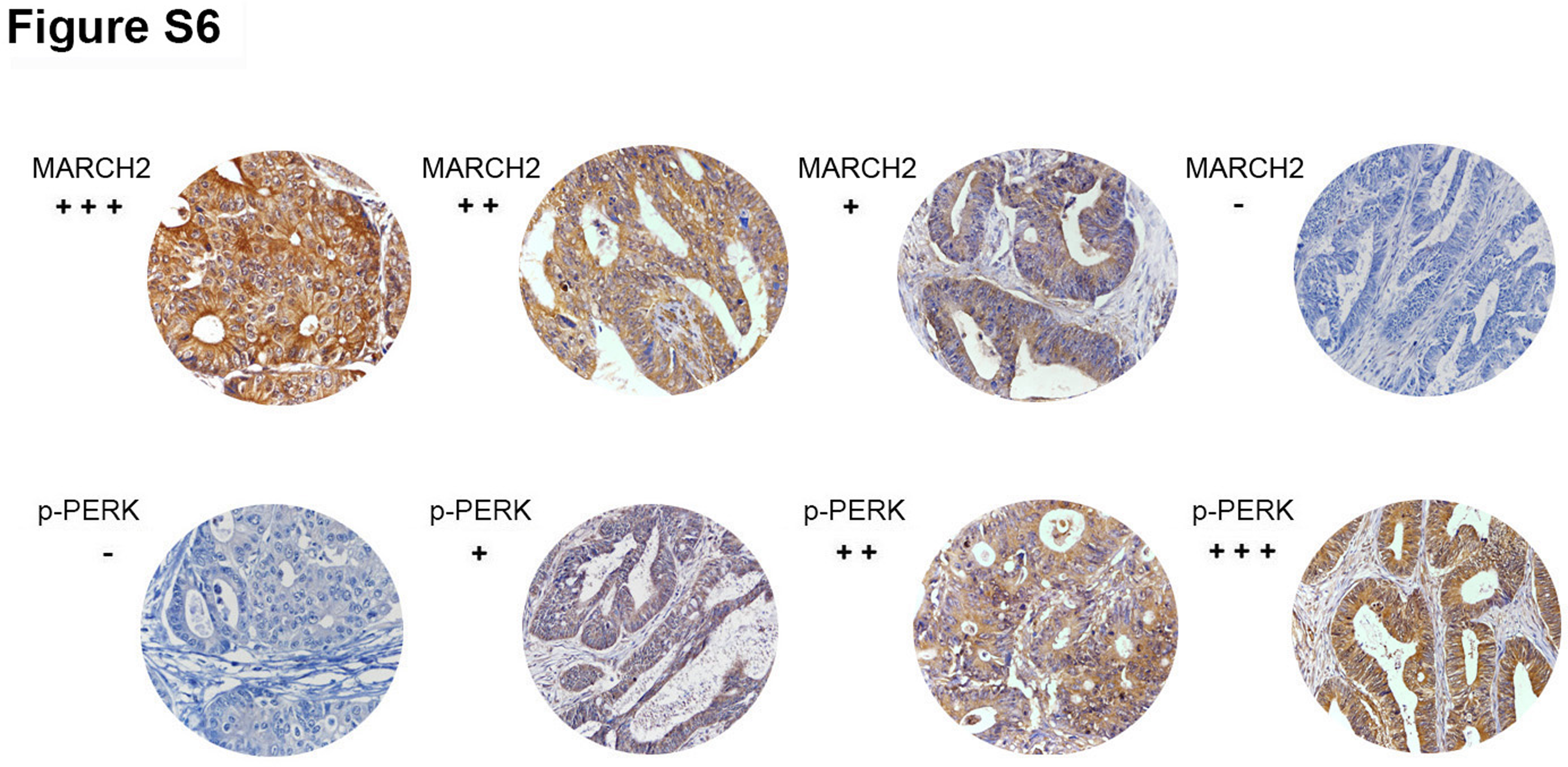

Supplement: Supplementary Figure 6 [file cddis2017347x7.tif]

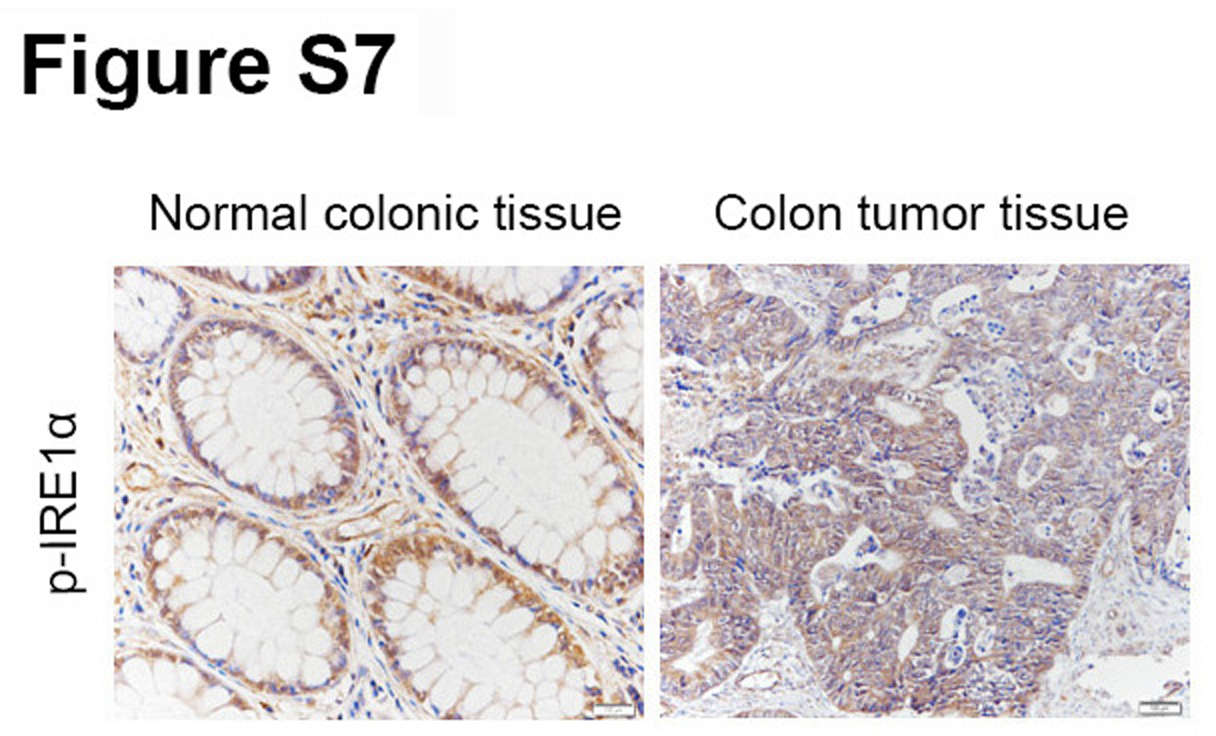

Supplement: Supplementary Figure 7 [file cddis2017347x8.tif]
